# Identification of Arp2/3 Complex Subunits as Prognostic Biomarkers for Hepatocellular Carcinoma

**DOI:** 10.3389/fmolb.2021.690151

**Published:** 2021-07-09

**Authors:** Shenglan Huang, Dan Li, LingLing Zhuang, Liying Sun, Jianbing Wu

**Affiliations:** ^1^ Department of Oncology, The Second Affiliated Hospital of Nanchang University, Nanchang, China; ^2^ Jiangxi Key Laboratory of Clinical and Translational Cancer Research, Nanchang, China; ^3^ Department of Gynaecology, The Second Affiliated Hospital of Nanchang University, Nanchang, China

**Keywords:** hepatocellular carcinoma (HCC), Arp2/3 complex subunits, prognosis, immune infiltration, clinical features

## Abstract

The actin-related protein 2/3 complex (Arp2/3) is a major actin nucleator that has been widely reported and plays an important role in promoting the migration and invasion of various cancers. However, the expression patterns and prognostic values of Arp2/3 subunits in hepatocellular carcinoma (HCC) remain unclear. In this study, The Cancer Genome Atlas (TCGA) and UCSC Xena databases were used to obtain mRNA expression and the corresponding clinical information, respectively. The differential expression and Arp2/3 subunits in HCC were analyzed using the “limma” package of R 4.0.4 software. The prognostic value of each subunit was evaluated using Kaplan–Meier survival analysis and Cox proportional hazards regression analyses. The results revealed that mRNA expression of Arp2/3 members (ACTR2, ACTR3, ARPC1A, APRC1B, ARPC2, ARPC3, ARPC4, ARPC5, and ARPC5L) was upregulated in HCC. Higher expression of Arp2/3 members was significantly correlated with worse overall survival (OS) and shorter progression-free survival (PFS) in HCC patients. Cox proportional hazards regression analyses demonstrated that ACTR3, ARPC2, and ARPC5 were independent prognostic biomarkers of survival in patients with HCC. The relation between tumor immunocyte infiltration and the prognostic subunits was determined using the TIMER 2.0 platform and the GEPIA database. Gene set enrichment analysis (GSEA) was performed to explore the potential mechanisms of prognostic subunits in the carcinogenesis of HCC. The results revealed that ACTR3, ARPC2, and ARPC5 were significantly positively correlated with the infiltration of immune cells in HCC. The GSEA results indicated that ACTR3, ARPC2, and ARPC5 are involved in multiple cancer-related pathways that promote the development of HCC. In brief, various analyses indicated that Arp2/3 complex subunits were significantly upregulated and predicted worse survival in HCC, and they found that ACTR3, ARPC2, and ARPC5 could be used as independent predictors of survival and might be applied as promising molecular targets for diagnosis and therapy of HCC in the future.

## Introduction

Liver cancer is the fourth leading cause of cancer-related mortality and ranks sixth in terms of incidence rate, and it is estimated that more than one million patients will die from liver cancer in 2030 (Akinyemiju et al., 2017; Villanueva, 2019). Hepatocellular carcinoma (HCC), which accounts for 75–80% of liver cancers, is the most common liver cancer, with its morbidity and prevalence increasing annually (Tan et al., 2003). Approximately 700,000 patients are newly diagnosed with HCC every year, with over half of the cases occurring in developing countries, and Asian countries account for three-quarters of HCC-related deaths (Asia-Pacific Working Part, 2010). This high fatality rate is mainly due to the low early diagnosis rate of HCC, rapid progress, fewer treatments for advanced cancer, particularly with high heterogeneity in cancer, undefined molecular mechanisms, and lack of early prognostic indicators. Therefore, it is imperative to search for highly sensitive and specific prognostic markers and potential drug targets to clarify the molecular mechanisms and improve the prognosis of HCC patients.

The actin-related protein 2/3 complex (Arp2/3) is a major actin nucleator that is responsible for promoting the nucleation of microfilaments, facilitating the assembly process of intracellular actin monomers into microfilaments, which forms the cellular structure and promotes processes involved in the formation of cell–cell junctions, motility of pathogens, and transport of vesicles (García-Ponce et al., 2015). The process of actin filament nucleation also plays an important role in the formation of invasive pseudopodia in cancer cells (Firat-Karalar and Welch, 2011). Abnormal migration and invasion are critical factors for tumor metastasis. Kiuchi et al. (Kiuchi et al., 2011) have shown that Arp2/3 is related to the formation of pseudopodia and the movement of bladder cancer cells. It has also been reported that high expression of Arp2/3 positively correlates with the malignancy of glioma specimens and that Arp2/3 system deregulation promotes cancer progression and directly impacts patient survival (Liu et al., 2013; Molinie and Gautreau, 2018). Thus, Arp2/3 plays a crucial role in tumor invasion and metastasis.

The actin-related protein 2/3 complex family (Arp2/3) consists of seven evolutionarily conserved subunits, including two actin-associated proteins, the Arp2 and Arp3 subunits (ACTR2 and ACTR3), and five accessory subunits, ARPC1, ARPC2, ARPC3, ARPC4, and ARPC5. ARPC1 has two subtypes in humans: ARPC1A and ARPC1B. ARPC5 subunits are classified into ARPC5 and ARPC5L (Abella et al., 2016). The center of the Arp2/3 complex is composed of ARPC2 and ARPC4, forming a C-type structure, while the other subunits interact around the center, forming a stable Arp2/3 complex, and ACTR2 and ACTR3 are in contact with the pointed end of the new daughter filament (Pollard and Beltzner, 2002). Many studies have shown that abnormal expression of Arp2/3 subunits is associated with the proliferation and invasion of various cancers, including pancreatic cancer (Rauhala et al., 2013), gastric cancer (Zhang et al., 2017a), colorectal cancer (Su et al., 2018), breast cancer (Chen et al., 2019; Cheng et al., 2019), bladder cancer (Chen et al., 2019; Cheng et al., 2019), gliomas (Liu et al., 2013), lung squamous cell carcinoma (lung SCC) (Moriya et al., 2012), and head and neck squamous cell carcinoma (HNSCC) (Kinoshita et al., 2012). However, the significance of the whole Arp2/3 subunit expression and the prognostic value of HCC has not yet been determined. Few studies have reported the correlation between the mRNA expression of Arp2/3 subunits and immune infiltration in HCC.

In this study, the mRNA expression and the prognostic values of the Arp2/3 family members were comprehensively evaluated in HCC according to updated public resources and multiple bioinformatics analyses. Furthermore, we investigated the potential correlation between Arp2/3 family members and immune cell infiltration levels in HCC.

## Materials and Methods

### Data Acquisition

The high-throughput sequencing (HTSeq) fragments per kilobase of transcript per million mapped reads (FPKM) data of hepatocellular carcinoma (HCC) tissues, including 374 tumor samples and 50 normal control samples, were downloaded from The Cancer Genome Atlas (TCGA) (https://portal.gdc.cancer.gov/), which is a free and available reference database for cancer research covering 33 cancer types, 20,000 primary cancer samples, and matched normal samples (Tomczak et al., 2015). The corresponding clinical information was obtained from the UCSC Xena database (http://xena.ucsc.edu/), which included the survival status, survival time (days), sex, age, histological grade, TNM stage, and progression-free survival (PFS) time of HCC patients. The mRNA expression data of HCC cell lines were obtained from the Cancer Cell Line Encyclopedia (CCLE) (https://portals.broadinstitute.org/ccle) (Barretina et al., 2012).

### Differential Expression of Arp2/3 Subunits at mRNA Levels in Pan-Cancers and Hepatocellular Carcinoma

First, the mRNA differential expression of Arp2/3 members in pan-cancers and corresponding normal tissues was analyzed using the Oncomine 4.5 database (https://www.oncomine.org/), which is an online large data–mining platform and integrated oncogene microarray database covering 715 datasets and 86,733 samples (Rhodes et al., 2007). In this study, the cell color is determined by the best gene rank percentile for the analyses within the cell; Student’s *t*-test was applied to calculate the *p* value for expression differences of Arp2/3 subunits between cancer and normal controls. The threshold parameters were set as follows: *p*-value < 0.0001, fold change = 1.5, and gene rank = 10%.

Thereafter, according to the RNA-Seq FPKM data of HCC downloaded from TCGA, the Arp2/3 subunits’ differential expression in HCC tissues compared to that in normal tissues was analyzed using the “limma” package of R 4.0.4 software (http:///www.r-project.org/). After removing some samples, the remaining 371 tumor samples and 50 normal samples were included in differential expression analysis. The Wilcox test was applied to generate *p*-values, statistical significance was set at *p* < 0.05, and the results were visualized using heatmaps. Moreover, UALCAN (Chandrashekar et al., 2017) (http://ualcan. path.uab.edu) was selected for further verification of the facticity of the differential expression results above, Student’s *t*-test was used to verify expression differences, and *p* < 0.05 indicates that the difference is statistically significant. Significantly differentially expressed subunits were selected for prognostic analysis.

Subsequently, we also explored whether the mRNA expressions of Arp2/3 subunits were correlated with each other in HCC tissues; Pearson’s correlation was performed using the “corrplot” R package, the Pearson product–moment correlation coefficient (Pearson’s R) represents the degree of correlation between the two subunits, and Pearson’s R > 0.4 was considered as statistical correlation.

Besides, we downloaded the mRNA expression data of the Arp2/3 subunits in HCC cell lines from the Broad Institute Cancer Cell Line Encyclopedia (CCLE) (https://portals.broadinstitute.org/ccle), which provides public access to genomic data, analysis, and visualization of over 1,100 cell lines (Chen et al., 2019; Cheng et al., 2019). The ggplot2 package of R 4.0.4 software was used to explore Arp2/3 subunit expression levels in different HCC cell lines.

### Differential Expression of Arp2/3 Subunits at the Protein Level in Hepatocellular Carcinoma Tissues

In addition to assessing the mRNA expression level of Arp2/3 members, protein expression analysis of Arp2/3 subunits was conducted using data from The Human Protein Atlas (HPA, https://www.proteinatlas.org/). The HPA provides typical immunohistochemistry profiling data for more than 8,000 patients and contains 26,941 antibodies targeting 17,165 unique proteins; it is also available for free download of nearly 20 common cancer types (Thul et al., 2017). In this study, the representative immunohistochemistry images of Arp2/3 members were downloaded directly from the HPA. We then compared the protein expression differences of Arp2/3 members in HCC and normal liver tissues.

### Prognostic Values of Arp2/3 Subunits in Hepatocellular Carcinoma

First, the mRAN expression value of Arp2/3 subunits and relevant clinical information of HCC were downloaded from the TCGA and UCSC Xena databases. After deleting some samples with incomplete follow-up information, we analyzed the remaining 370 HCC patients with complete overall survival time and 372 patients with progression-free survival information. Maximally selected rank statistics was used to determine an optimal cutoff value for each subunit, and the patients were divided into high- or low-expression groups according to the optimal cutoff value. Then Kaplan–Meier survival analysis and the log-rank test were performed to compare the overall survival (OS) or progression-free survival (PFS) difference between the two groups. Statistical significance was set at *p* < 0.05.

Furthermore, univariate and multivariate Cox regression analyses were conducted to explore whether the Arp2/3 subunits could be used as independent factors for the prognosis of HCC patients, integrating the following clinicopathological factors: age, sex, grade, and clinical stage. The results were presented with a hazard ratio (HR) and 95% confidence interval (CI), and statistical significance was set at *p* < 0.05. The subunits that significantly affected survival were chosen for further analyses.

### Clinicopathological Analysis of Arp2/3 Subunits in Hepatocellular Carcinoma

UALCAN (http://ualcan.path.uab.edu) is a comprehensive interactive web server based on TCGA RNA-seq and clinical data (Chandrashekar et al., 2017). In this study, UALCAN was used to assess the association between the mRNA expression of Arp2/3 subunits and the clinicopathological parameters in HCC patients, including individual cancer stages and the nodal metastasis status. The results were obtained directly from the UALCAN website (TCGA data processed by unified standards). Student’s *t*-test was used to verify expression differences, and statistical significance was set at *p* < 0.05.

### Immune Infiltration Analysis of the Arp2/3 Family in Hepatocellular Carcinoma

Tumor cells and tumor-infiltrating immune cells (TIICs) interact closely in cancer progression; thus, we investigated the connections between TIICs and Arp2/3 subunits using the TIMER 2.0 platform (http://timer.comp-genomics.org/), which provides a systematic analysis of the specific gene(s) and immune cell infiltration in different cancers, including 32 types of cancers and 10,897 samples from TCGA (Li et al., 2020). In this study, the TIICs included CD4+ T cells, CD8+ T cells, B cells, neutrophils, and macrophages. We further explored the association between Arp2/3 subunits and biomarkers of subsets of TIICs using GEPIA databases (Tang et al., 2017) (http://gepia.cancer-pku.cn/). TIMER 2.0 and GEPIA correlation analysis employed the Spearman test and *p* < 0.05 was considered statistically significant, and the correlation strength was evaluated using Spearman’s rank correlation Rho according to the previous studies, Rho 0.00–0.19 being “very weak,” Rho 0.20–0.39 being “weak,” Rho 0.40–0.59 being “moderate,” Rho 0.60–0.79 being “strong,” and Rho 0.80–1.0 being “very strong” (Lin et al., 2020).

### Gene Set Enrichment Analysis (GSEA)

To explore the potential biological mechanism by which Arp2/3 subunits affect the carcinogenesis and progression of HCC, the transcriptome data of HCC from TCGA were selected for gene set enrichment analysis (GSEA) using GSEA 4.1.0 software. The c2. cp.kegg.v7.4. symbols.gmt downloaded from the Molecular Signatures Database (http://www.gseamsigdb.org/gsea/msigdb/collections. jsp) was used as the reference. GSEA was executed using a random combination number of 1,000 permutations, and a false discovery rate (FDR q-value) <0.01 was used to identify the significantly enriched pathways.

### Statistical Analysis

Statistical analyses were performed using R software (https://www.r-project.org/, version 4.0.4). The HTSeq FPKM mRNA data from the TCGA database were disposed using Perl 5.30.0 software (https://www.perl.org/). The “limma” R package and the Wilcox test were used to analyze the different expressions of Arp2/3 subunits in HCC. Besides, Student’s *t*-test was used to verify expression differences in UALCAN. The “corrplot” R package was used for the correlation analysis of Arp2/3 members. Kaplan–Meier survival analysis and Cox proportional hazards regression analysis were conducted to assess the prognostic significance of Arp2/3 subunits. All statistical tests were two-sided, and statistical significance was set at *p* < 0.05.

## Results

### The mRNA Expression of Arp2/3 Subunits in Pan-Cancers and Hepatocellular Carcinoma

**FIGURE 1:**
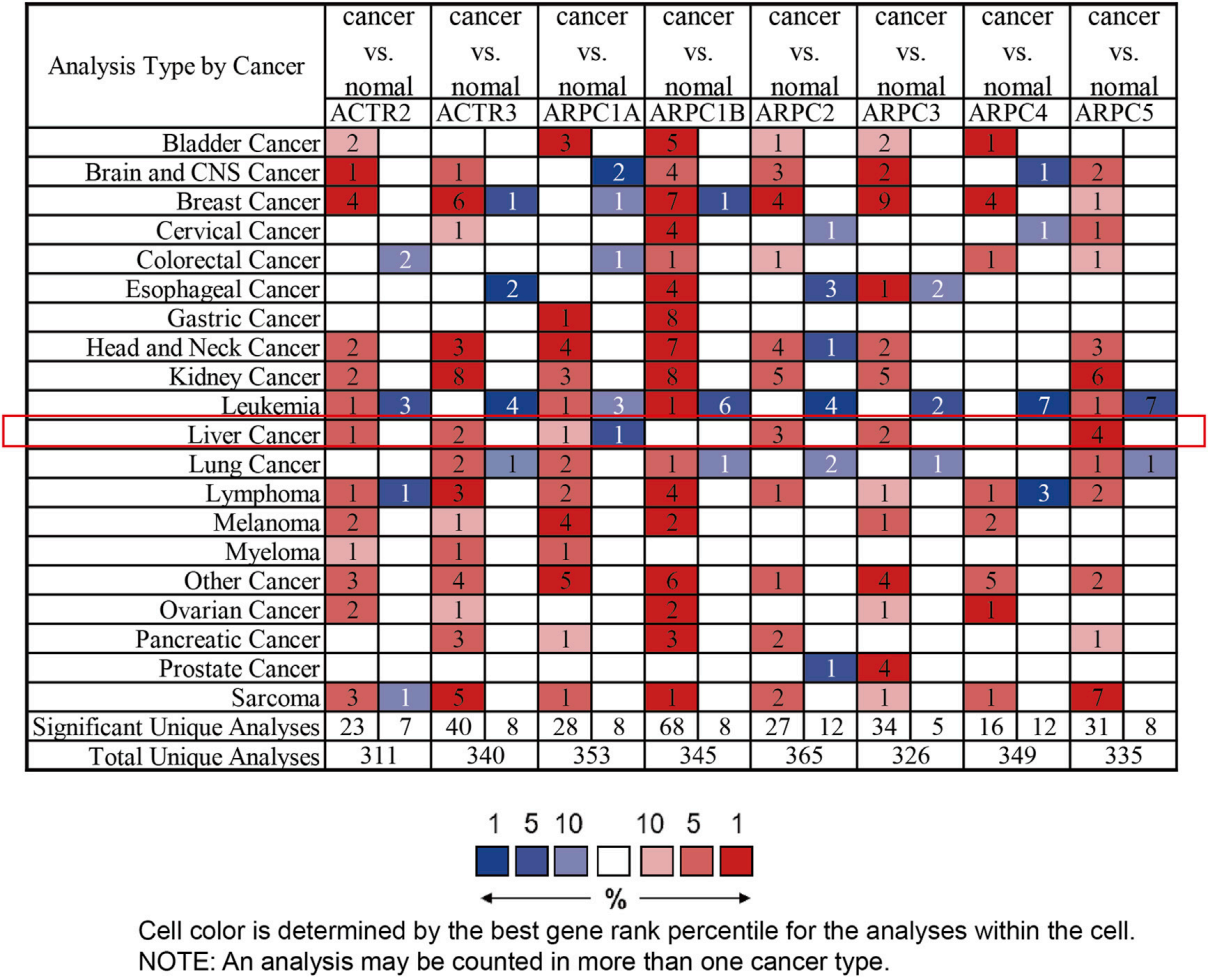
mRNA expression level of Arp2/3 complex members in different types of cancer from multiple datasets (the Oncomine database). Difference of transcriptional expression was compared using Student’s *t*-test and cutoff of parameters: *p*-value < 0.0001, fold change = 1.5, and gene rank = 10%. The red color in the cell represents overexpression, and green represents down-expression. The number in each cell presents the amount of datasets.

**FIGURE 2:**
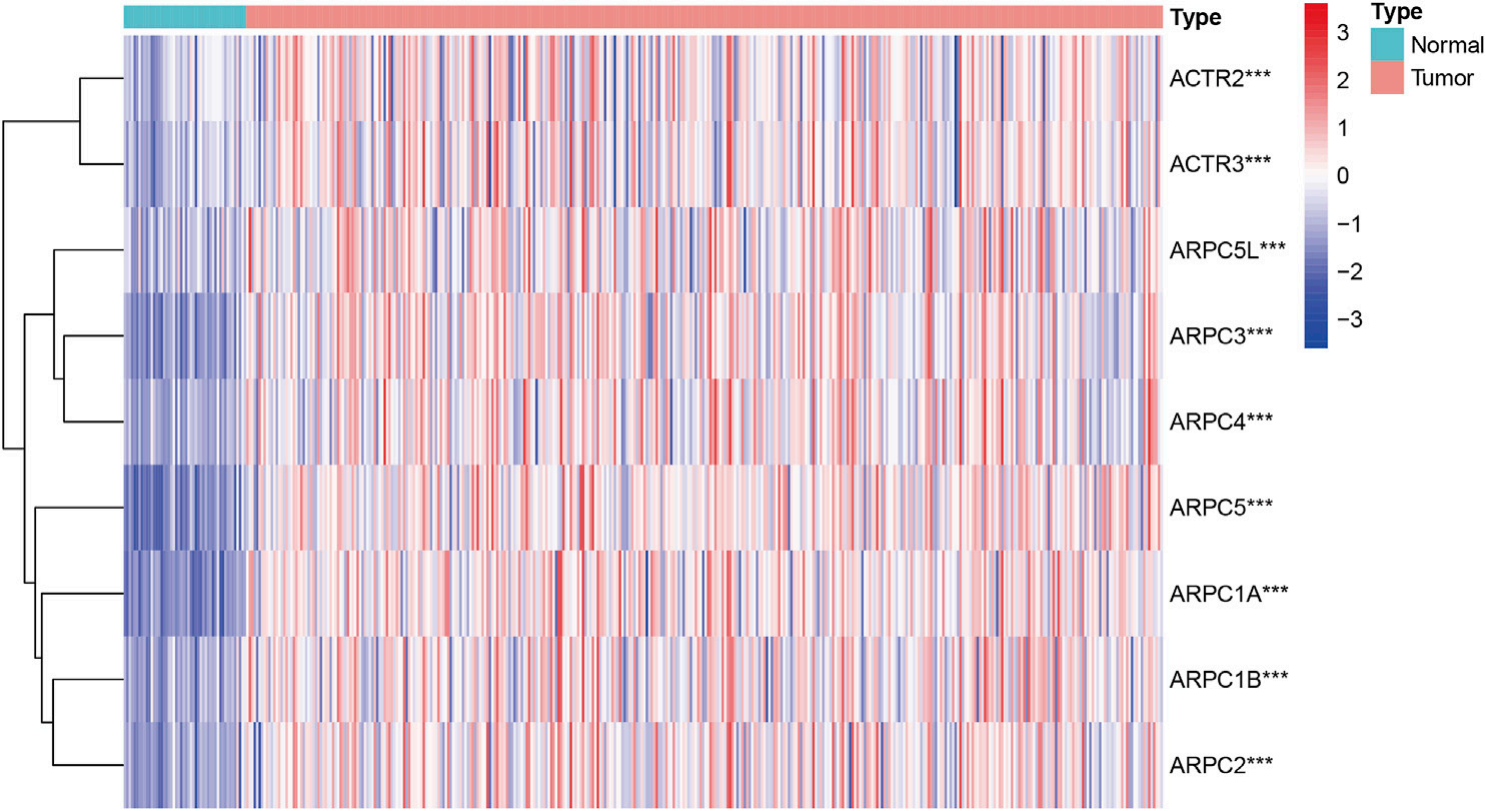
Expression profile level of Arp2/3 complex members in HCC tissues visualized using a heatmap. Red stands for overexpression, and blue represents down-expression. ***stands for *p* < 0.001.

**FIGURE 3:**
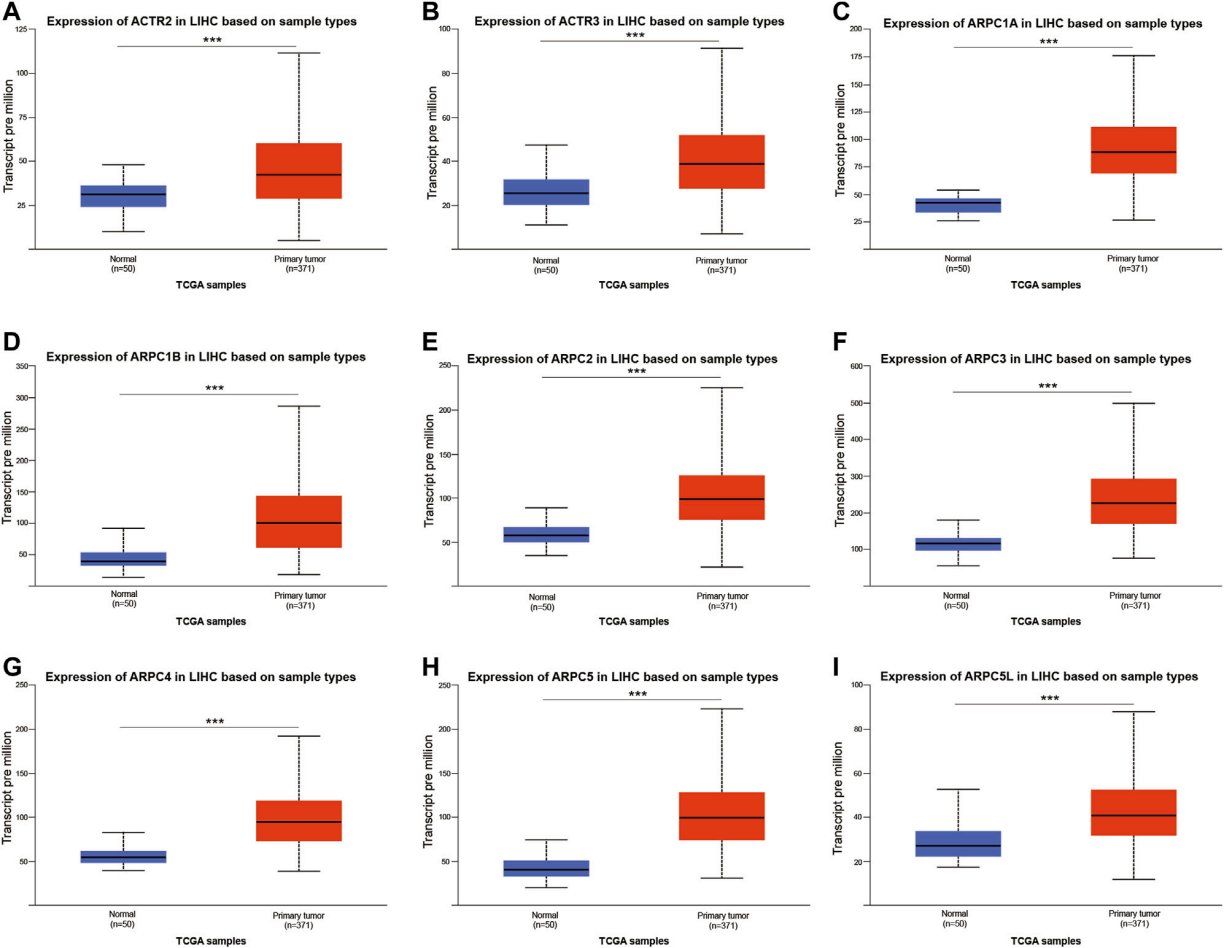
Relative expression of Arp2/3 complex members in normal tissues and HCC tissues based on the UALCAN database **(A–I)**, _***_stands for *p* < 0.001.

**FIGURE 4:**
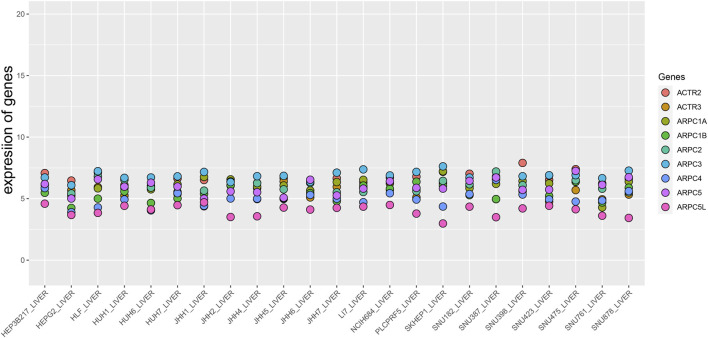
Expression level of Arp2/3 complex members in HCC cell lines (the CCLE database). The abscissa represents different HCC cell lines, and the ordinate is the expression value of genes.

First, the mRNA differential expression of Arp2/3 subunits in pan-cancers and the corresponding normal tissues was analyzed using the Oncomine database. As shown in Figure 1, overexpression of Arp2/3 subunits was observed in many kinds of cancers, including liver cancer. Based on the TCGA database, after collecting 371 HCC samples and 50 normal control samples, the mRNA differential expression of Arp2/3 subunits was obtained using the “limma” R package. We found that all of the Arp2/3 family members (ACTR2, ACTR3, ARPC1A, APRC1B, ARPC2, ARPC3, ARPC4, ARPC5, and ARPC5L) were significantly upregulated in HCC tissues (Figure 2). The differential expression analyzed using the UALCAN databases also showed that Arp2/3 subunits were upregulated in HCC tissues compared with normal control tissues, which was in accordance with the results above (Figures 3A–I). We used the CCLE databases to probe the mRNA expression of Arp2/3 subunits in HCC cell lines and found that Arp2/3 subunits were widely expressed in 23 HCC cell lines; among them, the expression levels of ACTR2 and ARPC3 were higher than those of other subunits, and the ARPC5L expression level was lowest in HCC cell lines (Figure 4).

**FIGURE 5:**
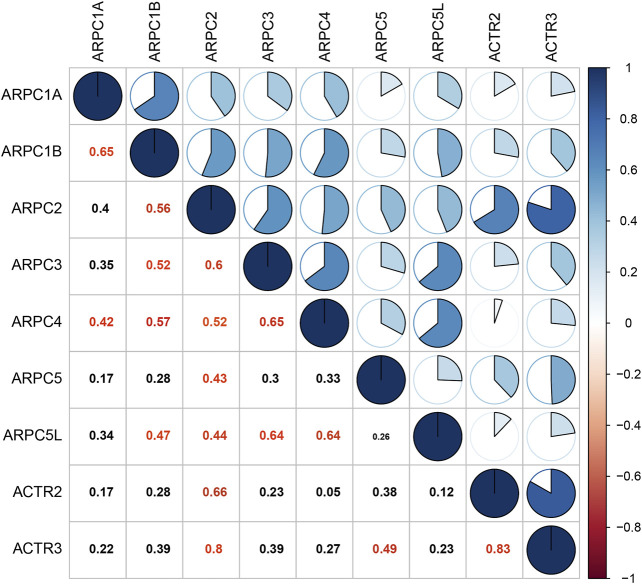
Correlation analysis of each Arp2/3 complex member. The data were analyzed by Pearson’s correlation, and Pearson’s R cutoff value was 0.4. The red values represent significant correlation between the Arp2/3 members.

Pearson’s correlation was performed to explore whether the mRNA expressions of Arp2/3 subunits were correlated with each other. The results revealed that the mRNA expressions of Arp2/3 subunits were correlated to a significant degree in HCC tissues, such as ARPC1A and ARPC1B (Pearson’s R = 0.65), ARPC2 and APRC3 (Pearson’s R was 0.6), ARPC2 and ACTR (Pearson’s R was 0.66), ARPC3 and ARPC4 (Pearson’s R was 0.65), ARPC3 and APRC5L (Pearson’s R was 0.64), ARPC4 and ARPC5L (Pearson’s R was 0.64), and ARTC2 and ARTC3 (Pearson’s R was 0.83), as shown in Figure 5.

### Protein Expression of Arp2/3 Subunits in Hepatocellular Carcinoma Tissue

**FIGURE 6:**
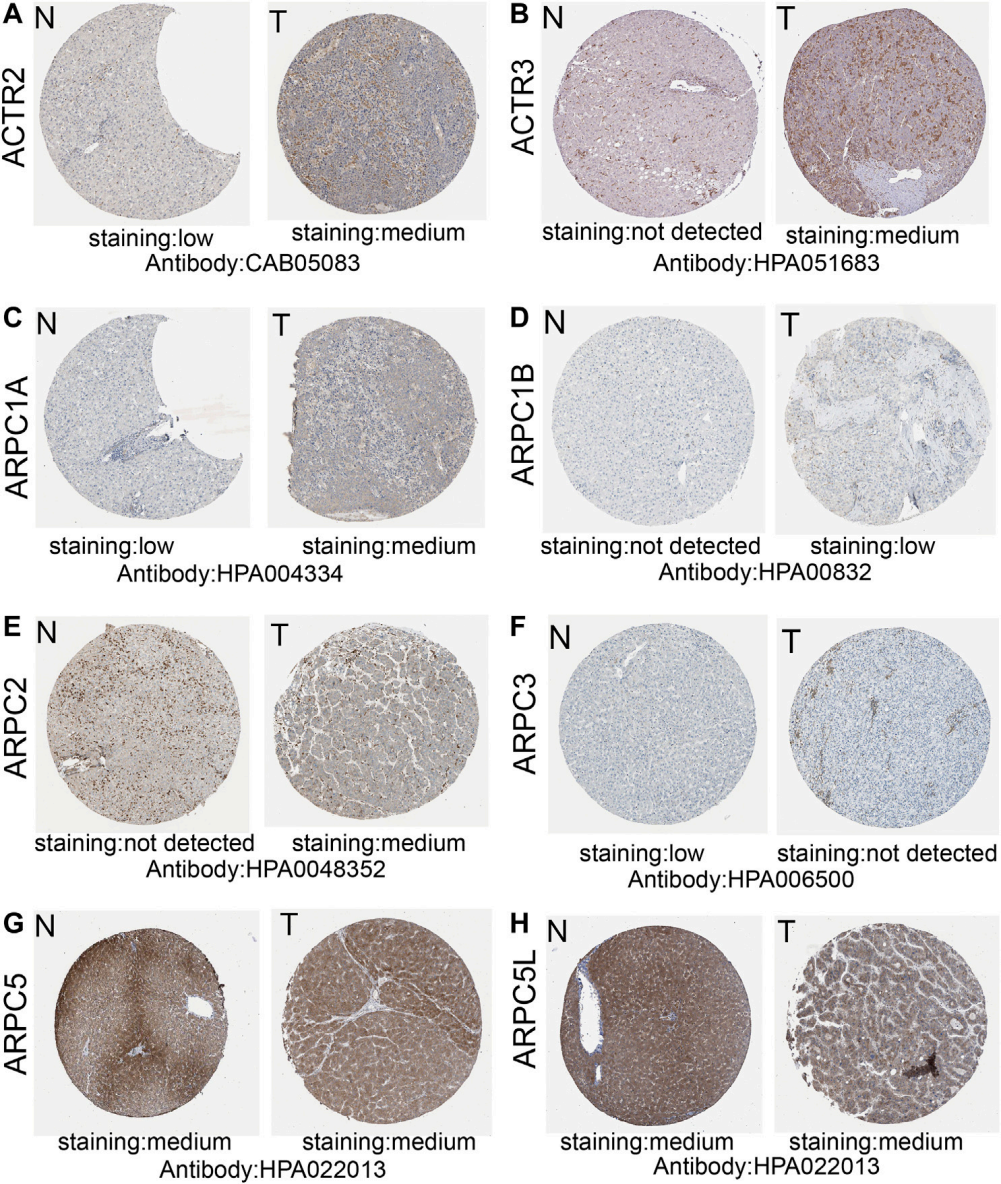
Representative immunohistochemistry images of Arp2/3 members (except for ARPC4) of HCC tissues and normal liver tissues in the Human Protein Atlas **(A–H)**. T, HCC tissues; N, normal liver tissues.

We obtained representative immunohistochemistry images from the Human Protein Atlas (HPA) to explore the protein expression conditions of Arp2/3 subunits in HCC. The results showed that higher protein expression of ACTR2, ACTR3, ARPC1A, ARPC1B, and ARPC2 was found in HCC tissues than in normal liver tissues, which have shown approximately the same results as the mRNA expression of Arp2/3 subunits. However, lower protein expression of ARPC3 was observed in HCC tissues than in normal tissues. There were no significant differences in the expression levels of ARPC5 and ARPC5L. Currently, there is no immunohistochemical map for ARPC4 detection in the HPA. The results of Arp2/3 subunit immunohistochemistry are shown in Figures 6A–H.

### Prognostic Values of Arp2/3 Subunits in Hepatocellular Carcinoma

**FIGURE 7:**
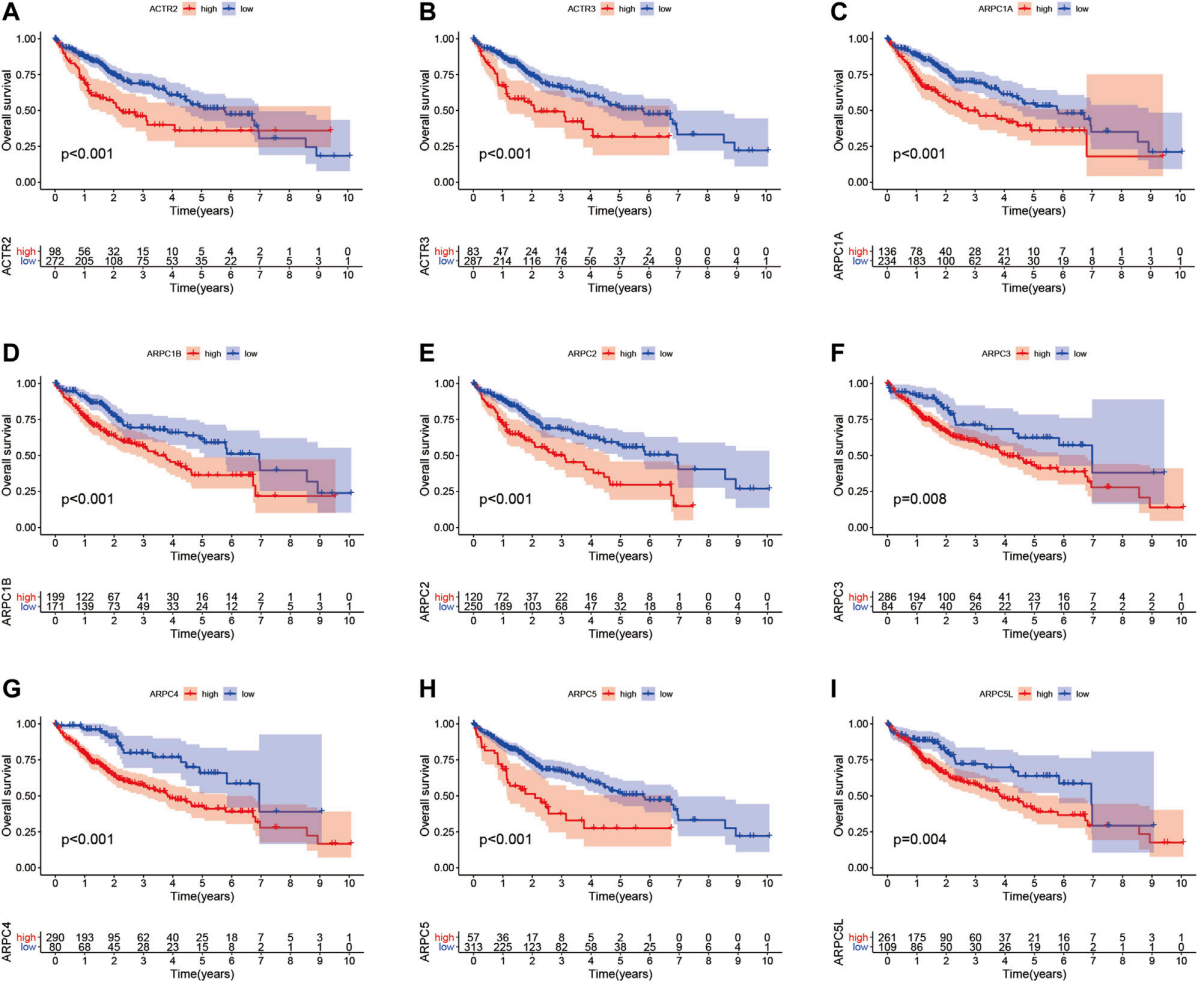
Kaplan–Meier survival analysis of the association between mRNA expression of Arp2/3 complex members and overall survival (OS) in HCC patients **(A–I)**. The information of HCC samples was derived from the TCGA and UCSC Xena databases.

**FIGURE 8:**
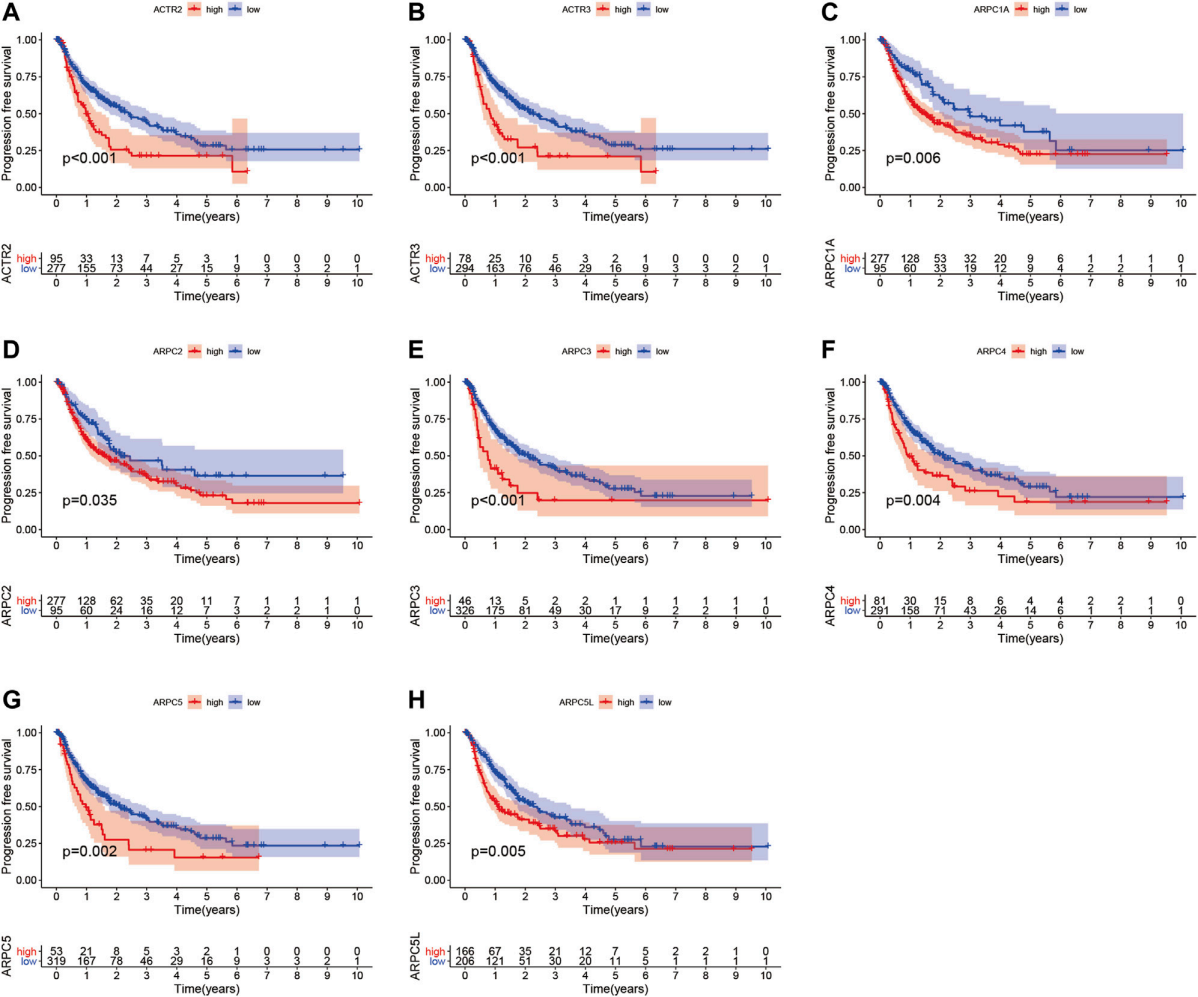
Kaplan–Meier survival analysis of the association between mRNA expression of Arp2/3 complex members and progression-free survival (PFS) in HCC patients **(A–H)**. The information of HCC samples was derived from the TCGA and UCSC Xena databases.

To examine the prognostic values of Arp2/3 subunits in HCC patients, we performed Kaplan–Meier survival analysis and the log-rank test. The Kaplan–Meier survival curves for overall survival (OS) indicated that HCC patients with high expression of ACTR2 (*p* < 0.001), ACTR3 (*p* < 0.001), ARPC1A (*p* < 0.001), ARPC1B (*p* < 0.001), ARPC2 (*p* < 0.001), ARPC3 (*p* = 0.008), ARPC4 (*p* < 0.001), ARPC5 (*p* < 0.001), and ARPC5L (*p* = 0.004) had worse OS than those with low expression, as shown in Figures 7A–I. The Kaplan–Meier survival curves for PFS showed that patients with high expression of ACTR2 (*p* < 0.001), ACTR3 (*p* < 0.001), ARPC1A (*p* = 0.0058), ARPC2 (*p* = 0.034), ARPC3 (*p* < 0.001), ARPC4 (*p* = 0.004), ARPC5 (*p* = 0.002), and ARPC5L (*p* = 0.005) had shorter PFS than those with low expression (Figures 8A–H). These results indicate that Arp2/3 subunits lead to poor prognosis in HCC patients.

**FIGURE 9:**
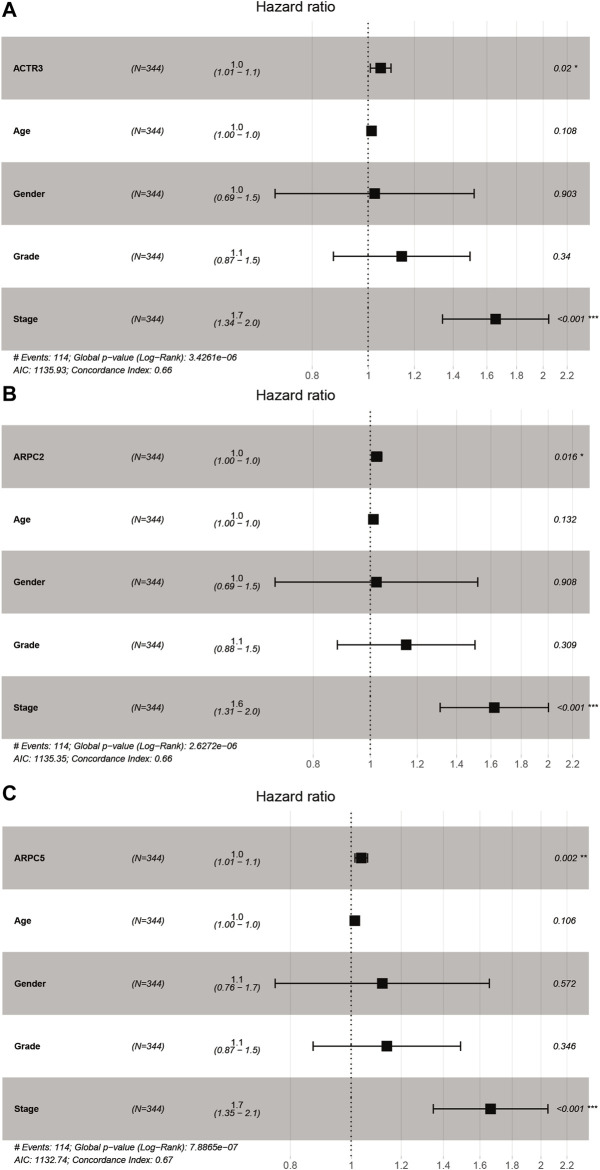
Forest plots of multivariate Cox regression analysis of Arp2/3 members with significant prognostic significance. **(A)** ACTR3; **(B)** ARPC2; and **(C)** ARPC5. *p < 0.05; **p < 0.01; ***p < 0.001.

Univariate and multivariate Cox proportional hazards regression analyses were carried out to evaluate whether the Arp2/3 subunits could be independently associated with HCC survival. The results of univariate Cox regression analysis showed that the Arp2/3 subunits (ACTR2, ACTR3, ARPC1A, ARPC2, ARPC3, ARPC4, ARPC5, and ARPC5L) and the clinical stage were associated with poor survival outcomes in HCC patients. The hazard ratio (HR), 95% confidence interval (CI), and *p* values are shown in Table 1. Multivariate Cox proportional hazards regression analysis revealed that the expressions of ACTR3 (HR = 1.0, 95%CI: 1.01–1.1, *p* = 0.002), ARPC2 (HR = 1.0, 95%CI: 1.00–1.0, *p* = 0.016), ARPC5 (HR = 1.0, 95%CI: 1.01–1.2, *p* = 0.002), and the clinical stage (HR = 1.6, 95%CI: 1.31–2.0, *p* < 0.001) were independent prognostic biomarkers of HCC survival, as shown in the forest plots in Figures 9A–C. Those results indicated that ACTR3, ARPC2, and ARPC5 are independently related to the prognosis of HCC patients and can be used as useful biomarkers to predict patients’ survival rate.

**TABLE 1:** Univariate Cox proportional hazards regression analyses of Arp2/3 members and clinical features in HCC.

Parameter	Univariate analysis		
	Hazard ratio	95% CI	*p* value
ARPC1A	1.011946	1.004–1.020	**0.002**
ARPC1B	1.004998	1.000–1.010	**0.053**
ARPC2	1.035206	1.016–1.055	**0.3E-03**
ARPC3	1.009771	1.002–1.017	**0.011**
ARPC4	1.014924	1.005–1.025	**0.004**
ARPC5	1.040619	1.017–1.064	**0.58E-03**
ARPC5L	1.048752	1.013–1.085	**0.006**
ACTR2	1.014755	1.004–1.026	**0.010**
ACTR3	1.064911	1.025–1.106	**0.001**
Age	1.010115	0.996–1.025	0.173
Gender	1.28922	0.883–1.882	0.188
Grade	1.133,154	0.881–1.457	0.330
Stage	1.679,735	1.369–2.062	**6.97E-07**

The bold values represent the factors that significantly associated with poor survival in HCC patients

### Correlation Analysis of Arp2/3 Subunits and Clinicopathological Features in Hepatocellular Carcinoma

**FIGURE 10:**
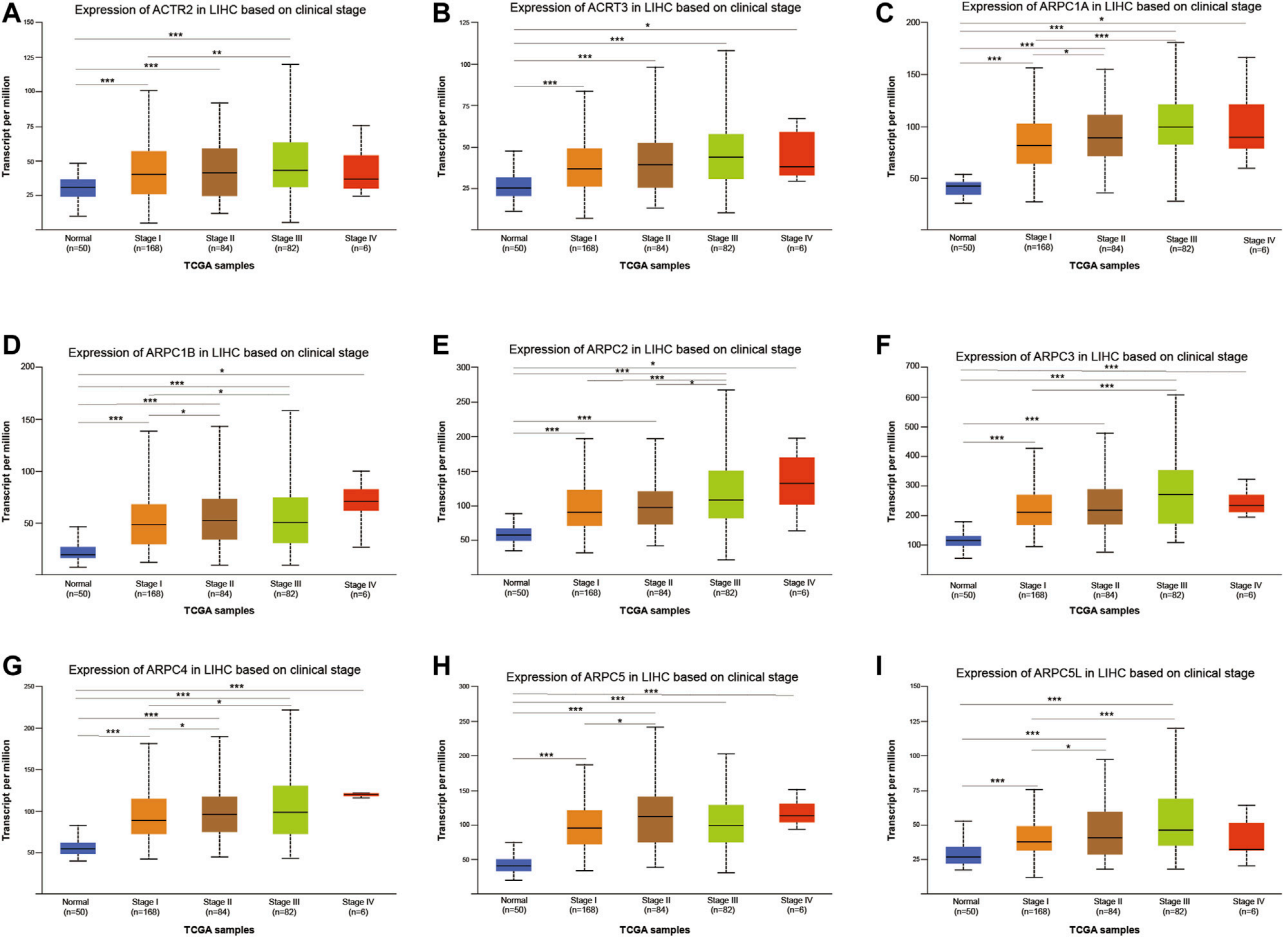
Analysis of association between mRNA expression of Arp2/3 members and cancer stages of HCC patients **(A–I)**. The mRNA expression of Arp2/3 members in normal individuals or in HCC patients of stages I, II, III, or IV. *p < 0.05; **p < 0.01; ***p < 0.001.

We used the UALCAN database to explore the relationship between the mRNA expression of Arp2/3 members and the clinicopathological parameters of HCC patients. As shown in Figures 10A–I, the results showed a definite association between the mRNA expression of Arp2/3 subunits (ACTR2, ACTR3, ARPC1A, ARPC1B, ARPC2, ARPC3, ARPC4, ARPC5, and ARPC5L) and cancer stages; patients with more advanced cancer stages tended toward higher mRNA expression of Arp2/3 subunits. The mRNA expression of Arp2/3 subunits (ACTR3, ARPC1A, ARPC1B, ARPC2, ARPC3, ARPC4, and ARPC5) was remarkably higher in HCC patients than in normal tissues. In contrast, there was no marked difference between stage IV and normal tissues in the mRNA expression of ACTR2, ACTR3, and ARPC5L, which may be due to the small sample size in stage IV (only six samples).

**FIGURE 11:**
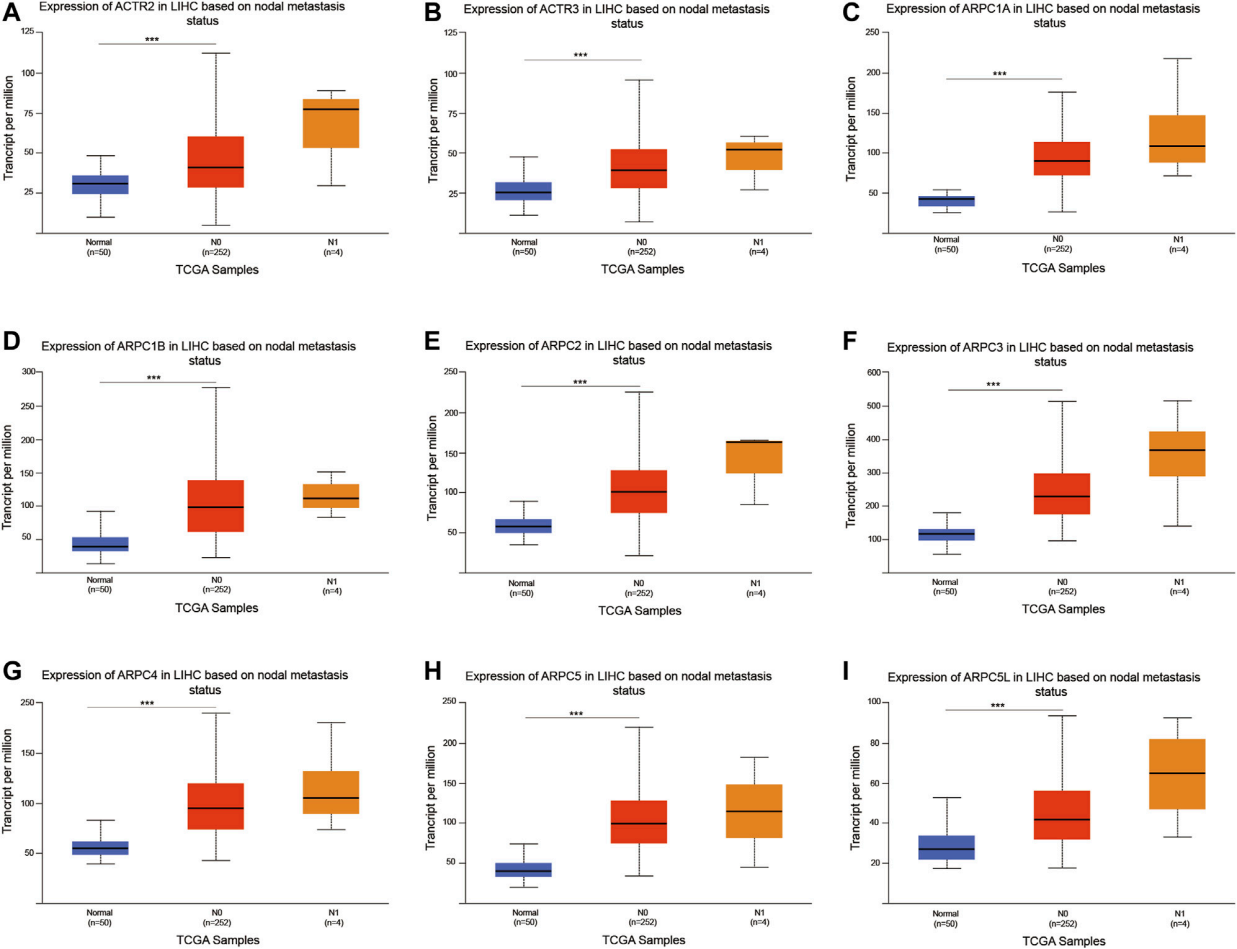
Analysis of association between mRNA expression of Arp2/3 members and the nodal metastasis status of HCC patients **(A–I)**. The mRNA expression of Arp2/3 members in normal individuals or in HCC patients of nodal metastasis status N0 or N1. *p < 0.05; **p < 0.01; ***p < 0.001.

We further investigated the relationship between the mRNA expression of Arp2/3 subunits and the nodal metastasis status in HCC patients. The results showed no significant relationship between the mRNA expression of Arp2/3 subunits and the nodal metastasis status (Figures 11A–I). This may be due to the small number of patients with lymph node metastasis in the TCGA database (n = 4). Nevertheless, the mRNA expression of Arp2/3 subunits (ACTR2, ACTR3, ARPC1A, ARPC1B, ARPC2, ARPC3, ARPC4, ARPC5, and ARPC5L) was remarkably higher in N0 patients than in those in the normal group.

### Association of Prognostic Arp2/3 Subunits With Immune Infiltration Level in Hepatocellular Carcinoma

**FIGURE 12:**
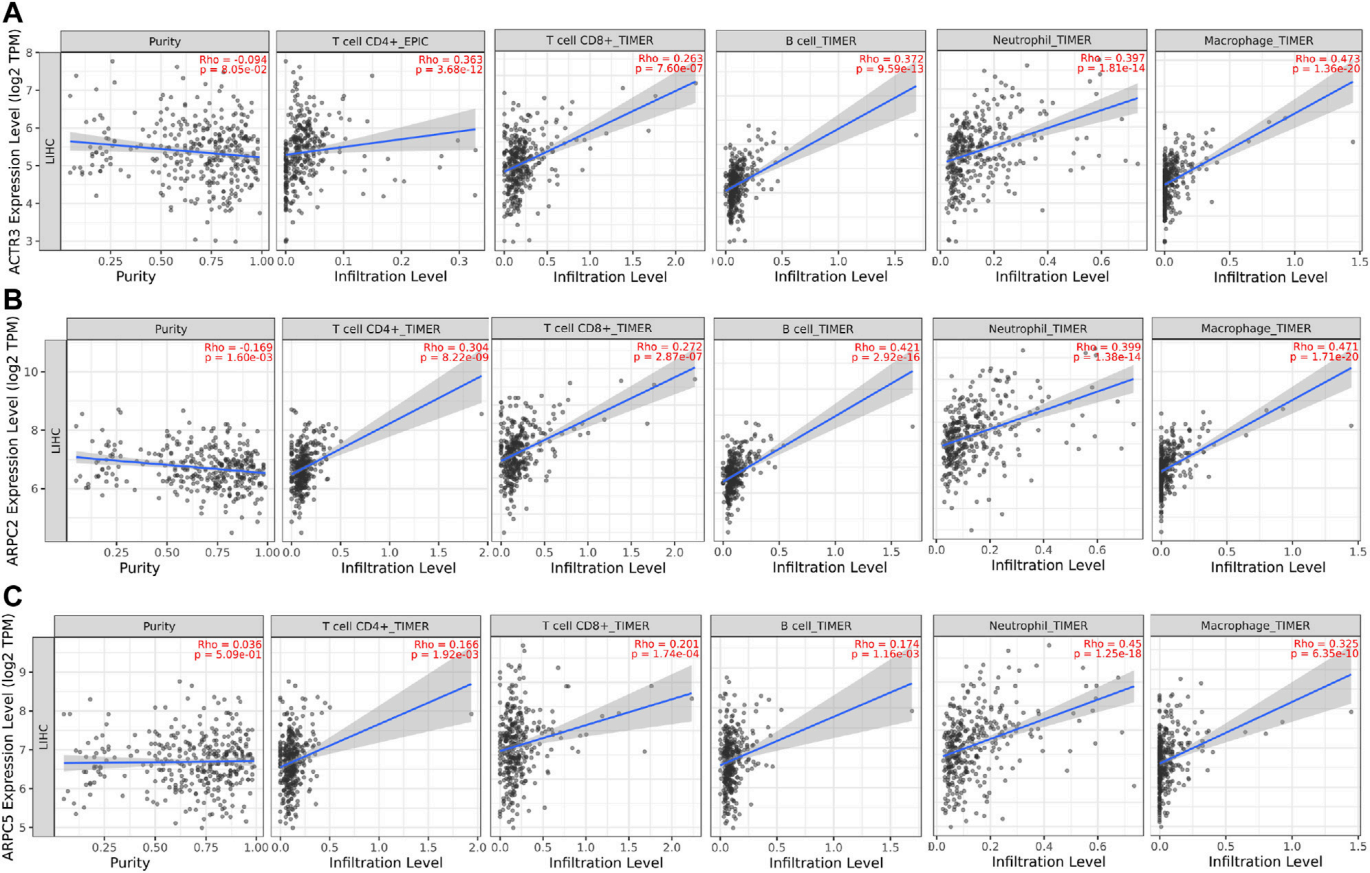
Correlation analysis between tumor-infiltrating immune cells (TIICs: CD4+ T cells, CD8^+^ T cells, B cells, neutrophils, and macrophages) and significant prognostic Arp2/3 members in HCC. **(A)** ACTR3; **(B)** ARPC2; and **(C)** ARPC5. Tumor purity is shown in the panels on the left.

Tumor-infiltrating immune cells (TIICs) in the tumor microenvironment (TME) play crucial roles in the tumorigenesis, progression, metastasis, and treatment resistance of tumors. To investigate the correlations between Arp2/3 subunits and TIICs, we first explored the associations between the independent prognostic biomarkers (ACTR3, ARPC2, and ARPC5) and the immune cells using the TIMER 2.0 platform. The results showed that the expression of ARPC2 was negatively correlated with tumor purity (Rho = -0.169, *p* = 1.6e-03), whereas the expression of ACTR3 and APRC5 was irrelevant to tumor purity. The expression levels of ACTR3, ARPC2, and ARPC5 were positively correlated with the immune infiltration of CD4+ T cells, CD8+ T cells, B cells, neutrophils, and macrophages, as shown in Figures 12A–C.

In addition, to investigate which types of TIIC subsets were correlated with prognostic Arp2/3 subunits, we analyzed the co-expression relationship of prognostic subunits and typical biomarkers of TIICs using the GEPIA database. As shown in Table 2, the higher expression of ACTR3 and ARPC2 was positively correlated with the expression of biomarkers of TIIC subsets, including B cells, CD8+ T cells, Th1 cells, Th2 cells, Th17 cells, Treg cells, neutrophils, M1 macrophages, and M2 macrophages. ACTR3 expression in HCC was significantly correlated with STAT1 (Th1), STAT6 (Th2), STAT3 (Th17), CCR8 (Treg), CDb11(neutrophils), PTGS2, and IRF5 (M1 macrophages). There was also a significant positive correlation between the expression of ARPC2 and TIIC biomarkers, including STAT1 and TNF (Th1), GATA3 and STAT5A (Th2), CCR8 and TGFB1(Treg), CDb11(neutrophils), PTGS2 and IRF5(M1 macrophages), and VSIG4 and MS4A4A (M2 macrophages). Higher expression of ARPC5 was positively correlated with most biomarkers of TIIC subsets. Some of these genes were moderately correlated with the expression of ARPC5, including STAT1(Th1), STAT6, STAT5A (Th2), STAT3(Th17), CDb11(neutrophils), and IRF5(M1 macrophages). In addition, the correlation between ARPC2, ARPC5, and ACTR3 and the other biomarkers of TIIC subsets was weak or irrelevant. The above results indicate that Arp2/3 genes might positively modulate the infiltration and activation of TIICs in HCC.

**TABLE 2:** Correlations between significant prognostic Arp2/3 subunits’ expression and biomarker expression of subsets of TIICs in HCC.

Types of TIICs	Gene markers	ARPC2		ARPC5		ACTR3	
		R	*P*	R	*P*	R	*P*
**B cell**	CD19	0.36	1.2E-12	0.18	5.3E-04	0.17	0.0012
	CD79A	0.34	3.6E-11	0.088	0.092	0.17	0.0012
**CD8**	CD8A	0.39	9.7E-15	0.031	0.55	0.23	6.3E-06
	CD8B	0.36	6.8E-13	0.11	0.038	0.12	0.027
**Th1**	TBX21	0.3	4.3E-09	0.11	0.032	0.19	1.7E-04
	STAT4	0.39	6.1E-15	0.19	2.9E-04	0.26	6.6E-07
	STAT1	**0.6**	**0.3E-37**	**0.46**	**5.3E-21**	**0.59**	**3.9E-36**
	TNF	**0.48**	**7E-23**	0.23	7.1E-06	0.37	1.3E-13
	IFNG	0.34	2.7E-11	0.13	0.012	0.17	0.0014
**Th2**	GATA3	**0.48**	**1.4E-22**	0.19	2.3E-04	0.38	2.6E-14
	STAT6	0.29	8.6E-9	**0.48**	**2.2E-22**	**0.57**	**1.6E-33**
	IL-13	0.11	0.043	0.055	0.29	0.12	0.019
	STAT5A	**0.54**	**7.4E-30**	**0.46**	**2.6E-20**	**0.48**	**3.2E-22**
**Th17**	STAT3	0.37	1.7E-13	**0.48**	**4.2E-23**	**0.63**	**2.1E-42**
	IL-17A	0.052	0.32	0.089	0.089	0.14	0.007
**Treg**	FOXP3	0.2	1E-04	0.12	0.018	0.26	4.8E-07
	CCR8	**0.55**	**6E-30**	0.36	5.1E-13	**0.56**	**2.9E-31**
	TGFB1	**0.56**	**1.2E-31**	0.23	6.4E-06	0.29	2.2E-08
**Neutrophils**	CD11b	**0.52**	**2.6E-27**	**0.47**	**1.8E-21**	**0.48**	**1.1e-22**
	CCR7	0.36	5E-13	0.18	5.4E-04	0.27	1.7e-07
	CD66b	0.13	0.014	−0.0051	0.92	0.1	0.054
**M1 macrophages**	NOS2	0.13	0.011	0.32	3.1E-10	0.35	2.4E-12
	PTGS2	**0.47**	**6.4E-22**	0.3	4.8E-09	**0.47**	**1E-21**
	IRF5	**0.44**	**2.9E-19**	**0.51**	**5.9E-26**	**0.46**	**1.8E-20**
**M2 macrophages**	CD163	0.33	5.7E-11	0.23	8E-06	0.15	0.003
	VSIG4	**0.47**	**3E-21**	0.27	1.7E-7	0.33	4.2E-11
	MS4A4A	**0.46**	**1.2E-20**	0.25	1.9E-6	0.36	4.6E-13

R, Spearman’s rank correlation Rho; *P*, *p* value. The bold values represent the correlation strength was above "moderate" between Arp2/3 subunits and biomarkers of subsets of TIICs.

### Potential Action Mechanism of Prognostic Arp2/3 Subunits in Hepatocellular Carcinoma Carcinogenesis

**FIGURE 13:**
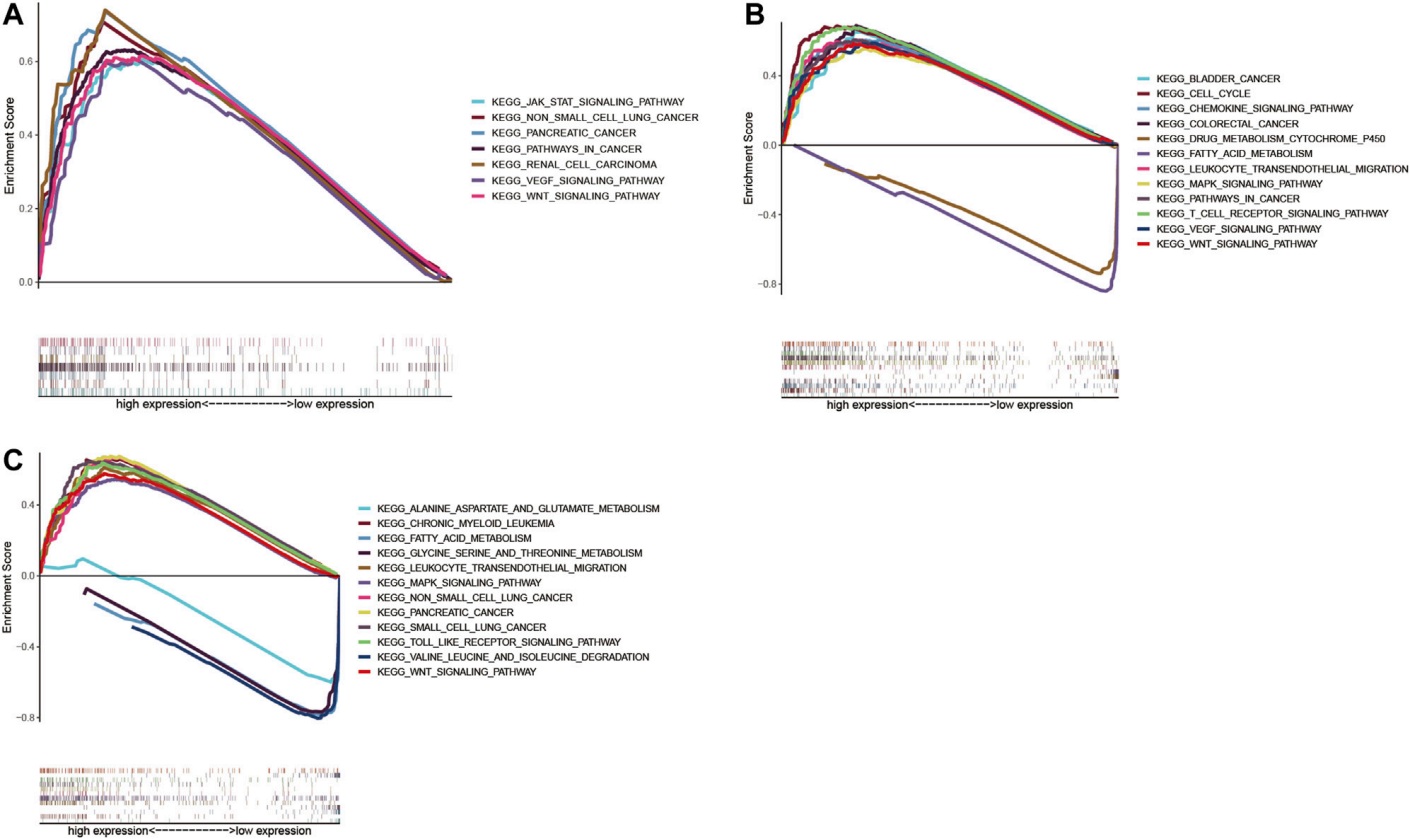
Gene set enrichment analysis (GSEA) of significant prognostic Arp2/3 subunits in HCC based on the cancer-related Kyoto Encyclopedia of Genes and Genomes (KEGG). **(A)** ACTR3; **(B)** ARPC2; and **(C)** ARPC5.

We identified ACTR3, ARPC2, and ARPC5 as independent prognostic biomarkers affecting the survival of HCC, and GSEA analysis was conducted to explore the potential biological mechanism by which Arp2/3 subunits lead to poor survival. According to the GSEA results, high expression of ACTR3 was positively related to 82 gene sets at FDR <0.01, the functions of which focused on regulation of the actin cytoskeleton, protein ubiquitination, the immune system process, genesis and progression of various tumors, leukocyte migration, and the DNA metabolic process; the ACTR3 overexpression was closely relevant to the “JAK-STAT signaling pathway,” the “WNT signaling pathway,” the “pathway in cancer,” the “VEGF signaling pathway,” “non–small-cell lung cancer,” “pancreatic cancer,” and “renal cell carcinoma,” as shown in Figure 13A. The GSEA results also indicated that high expression of ARPC2 was significantly positively related to 59 gene sets at FDR <0.01. Among them, the cancer-related pathways included the “WNT signaling pathway,” the “cell cycle,” the “pathway in cancer,” “bladder cancer,” “colorectal cancer,” the “VEGF signaling pathway,” the “MAPK signaling pathway,” and the “chemokine signaling pathway.” Besides, the “T cell receptor signaling pathway” and “leukocyte transendothelial migration” might be associated with immune cell infiltration (Figure 13B). Fourteen gene sets were significantly negatively related to the expression of ARPC2 at FDR <0.01, the functions of which focused on fatty metabolism, amino acid metabolism, and metabolism of xenobiotics by cytochrome P450 (Figure 13B). High expression of ARPC5 was significantly positively related to 43 gene sets at FDR <0.01, and the following pathways might be involved in tumor development and pathogenesis: the “MAPK signaling pathway,” “non–small-cell lung cancer,” “small-cell lung cancer,” “pancreatic cancer,” the “WNT signaling pathway,” and the “toll-like receptor signaling pathway” (Figure 13C). Five gene sets were significantly negatively related to the expression of ARPC2 at FDR <0.01, including fatty metabolism and amino acid metabolism (Figure 13C).

## Discussion

The actin-related protein 2/3 complex (Arp2/3) was first isolated from *Acanthamoeba* as an affinity complex for intracellular profibrin. It plays an important role in the formation of microfilaments and is related to cell movement. Recently, increasing numbers of studies have shown that Arp2/3 subunits are upregulated in various cancer tissues or cells involved in the proliferation, invasion, and metastasis of cancer. It was reported that the expression of Arp2 was significantly higher in cases with a high histologic grade and lymph node metastasis in adenocarcinomas of the lung and breast carcinoma (Semba et al., 2006; Iwaya et al., 2007). Eeva et al. (Laurila et al., 2009) found that ARPC1A acts as a novel regulator of cell migration and invasion in pancreatic cancer and has been suggested as a potential target for cancer anti-metastasis therapy. Zhang et al. (Zhang et al., 2017a) verified that ARPC2 expression was higher in gastric cancer tissues than in normal tissues and promoted gastric cancer cell proliferation and metastasis. It was reported that ARPC2 inhibitors, such as benproperine and pimozide, inhibited tumor invasion and metastasis of cancer cells in animal models (Choi et al., 2019). Similarly, significant overexpression of ARPC4 has also been observed in pancreatic carcinoma and gastric carcinoma, indicating a close association between APRC4 expression and tumor migration and invasion (Rauhala et al., 2013), (Kang et al., 2016). Furthermore, Xu et al. (Xu et al., 2020) have shown that ARPC4 is necessary for proliferation, migration, invasion, and pseudopodia formation in bladder cancer cells, suggesting that ARPC4 is a potential prognostic biomarker in these diseases. ARPC5 may function as an oncogene in the development of lung squamous cell carcinoma (lung SCC) and head and neck squamous cell carcinoma (HNSCC) and contributes to cancer cell migration and invasion, which is directly regulated by miRNA (Moriya et al., 2012), (Kinoshita et al., 2012). Arp2/3 complex silencing mediated by siRNA led to a reduction in the migration of pancreatic cells (Rauhala et al., 2013). In this study, we identified that ARP2/3 members were significantly overexpressed in various cancers, including HCC. Differential expression of ACTR2, ACTR3, ARPC1A, ARPC1B, and ARPC2, both at the mRNA and protein levels, was observed in patients with HCC. Moreover, there was a significant correlation between the expressions of each subunit, suggesting that ARP2/3 subunits may serve as potential biomarkers for HCC.

Furthermore, we evaluated the prognostic roles of Arp2/3 subunits in HCC using Kaplan–Meier survival analysis and Cox proportional hazards regression. We found that higher expression of ARP 2/3 subunits was associated with worse OS, and overexpression of ACTR2, ACTR3, ARPC1A, ARPC2, ARPC3, ARPC4, ARPC5, and ARPC5L was related to shorter PFS in HCC. We also identified that the expression of ACTR3, ARPC2, and ARPC5 was associated with poor survival of HCC patients as independent prognostic biomarkers. In addition, we found a specific correlation between the mRNA expression of the nine Arp2/3 members and the cancer stages of HCC. A previous study reported that ARPC2 was closely associated with the stage, nodal metastasis, and overall survival in breast cancer and that the TGF-β/EMT pathway is involved in ARPC2-mediated carcinogenesis (Cheng et al., 2019). Furthermore, ARPC2 was significantly associated with large tumor size, lymph node invasion, and high tumor stage *via* association analysis of 110 gastric cancer tissues, and ARPC2-positive patients exhibited lower RFS and OS rates than ARPC2-negative patients with gastric cancer (Zhang et al., 2017a). In addition, the ARPC5 high-expression group was associated with poor overall survival compared to that in the ARPC5 low-expression group, and multivariable analysis indicated that ARPC5 was an independent prognostic factor in patients with multiple myeloma (MM) (Xiong and Luo, 2018). Therefore, our results are consistent with those of previous studies on other tumors.

Cancer immunotherapy has caused significant breakthroughs in various malignancies. However, only a minority of patients with HCC respond to immunotherapy (Zhu et al., 2018). This is primarily due to the high heterogeneity of tumors, various immune microenvironments, lack of immune cell infiltration, and absence of predictive markers. Therefore, this study explored the association between the expression of prognostic genes and immune cell infiltration. The Arp2/3 complex is critical for chemotaxis and hagocytosis and is required for macrophage integrin effects and monocyte recruitment functions (Rotty et al., 2017). There is a strong relationship between the Arp2/3 complex and immune cells; for example, leukocytes need to adhere to cells to form synapses that kill infected cells, which literally squeeze their cell body during blood extravasation and efficiently migrate to the inflammatory focus (Tur-Gracia and Martinez-Quiles, 2021). Moreover, the cytoskeleton is crucially important for adhesive contact and migration in the development process of immune cells (Tur-Gracia and Martinez-Quiles, 2021). Since the Arp2/3 subunits assemble into a complex, the abnormity of one subunit is likely to affect the whole complex function. ARPC2 knockout mice have been reported to cause a dramatic decrease in peripheral T cell numbers and impaired T cell homeostasis, which was caused by a reduction in the surface TCR levels of T cells. There is a higher transcription level of ARPC2 in peripheral T cells than in thymocytes, and Arp2/3 complex–promoted actin nucleation is essential for peripheral T cell homeostasis (Zhang et al., 2017b). This study found that the expression of ACTR3, ARPC2, and ARPC5 was positively related to the immune infiltration of CD4+ T cells, CD8+ T cells, B cells, neutrophils, and macrophages in the HCC microenvironment. In addition, we discovered that ACTR3, ARPC2, and ARPC5 expression was significantly correlated with the biomarkers of CD4^+^ T cells, CD8+ T cells, neutrophils, M1 macrophages, and M2 macrophages. The above results indicate that Arp2/3 subunits participate in the activation and recruitment of TIICs in HCC and play a dual role in tumor immunity, which promotes antitumor immune cell infiltration and recruits immunosuppressive cells. Thus, further research is needed to help us understand the role of Arp2/3 subunits and tumor-related immune cell functions and, consequently, contribute to the application of immunotherapy.

Currently, few studies have been conducted on the specific mechanism by which ARP2/3 members promote tumor development and metastasis. Zhang et al. (Zhang et al., 2017a) found that oncogenic genes, including CTNND1, EZH2, BCL2L2, CDH2, VIM, and EGFR, were upregulated by ARPC2, and tumor suppressor genes PTEN, BAK, and CDH1 were downregulated by ARPC2. In breast cancer, ARPC2 expression significantly upregulated the expression of vimentin, N-cadherin, MMP-9, ZEB1, and MMP-3, activated the TGF-β pathway, and eventually led to epithelial–mesenchymal transition (EMT) (Cheng et al., 2019). Using gene set enrichment analysis (GSEA), this study found that Arp2/3 subunits mainly participate in regulating various cancer pathways, including colorectal cancer, pancreatic cancer, bladder cancer, lung cancer, renal cell carcinoma, and the VEGF, MAPK, and Wnt signaling pathways. In addition, leukocyte transendothelial migration and T cell receptor signaling pathways may increase immune cell infiltration. A validation study regarding the exact mechanism of ACTR2, ARPC2, and ARPC5 should be performed to confirm the above results further.

Although we discussed the important role of Arp2/3 complex members in HCC, the limitations of this study should be noted. First, the Arp2/3 subunits were analyzed and evaluated using limited data and clinical information from the genomic commons data. Moreover, protein differential expression of Arp2/3 subunits was presented with immunohistochemistry images from different samples, which may affect the results to some extent. It is preferable to collect a specific number of clinical samples and compare subunit expression levels in tumor tissues and adjacent noncancerous tissues. In addition, it is critical to verify the functional features and molecular mechanisms of Arp2/3 subunits using biological experiments and clinical research.

## Conclusion

This study systematically analyzed the expression profile and prognostic values of Arp2/3 complex members in HCC and found that ACTR3, ARPC2, and ARPC5 could be used as independent predictors of survival and might be applied as promising molecular targets for diagnosis and therapy of HCC in the future.

## Data Availability

The datasets presented in this study can be found in online repositories. The names of the repository/repositories and accession number(s) can be found in the article/Supplementary Material.

## Abbreviations

Arp2/3, actin‐related protein 2/3 complex; CCLC, Broad Institute Cancer Cell Line Encyclopedia; FPKM, fragments per kilobase of transcript per million mapped reads; GSEA, gene set enrichment analysis; HCC, hepatocellular carcinoma; HPA, The Human Protein Atlas; KEGG, Kyoto Encyclopedia of Genes and Genomes; OS, overall survival (OS); PFS, progress free survival; TCGA, The Cancer Genome Atlas; TIICs, tumor-infiltrating immune cells.

## References

[B1] Abella J. V. G. Galloni C. Pernier J. Barry D. J. Kjær S. Carlier M.-F. (2016). Isoform Diversity in the Arp2/3 Complex Determines Actin Filament Dynamics. Nat. Cel Biol 18 (1), 76–86. 10.1038/ncb3286 26655834

[B2] Akinyemiju T. Akinyemiju T. Abera S. Ahmed M. Alam N. Alemayohu M. A. (2017). The Burden of Primary Liver Cancer and Underlying Etiologies from 1990 to 2015 at the Global, Regional, and National Level: Results from the Global Burden of Disease Study 2015. JAMA Oncol. 3 (12), 1683–1691. 10.1001/jamaoncol.2017.3055 28983565 PMC5824275

[B3] Asia-Pacific Working Party on Prevention of Hepatocellular C (2010). Prevention of Hepatocellular Carcinoma in the Asia-Pacific Region: Consensus Statements. J. Gastroenterol. Hepatol. 25 (4), 657–663. 10.1111/j.1440-1746.2009.06167.x 20492323

[B4] Barretina J. Caponigro G. Stransky N. Venkatesan K. Margolin A. A. Kim S. (2012). The Cancer Cell Line Encyclopedia Enables Predictive Modelling of Anticancer Drug Sensitivity. Nature 483 (7391), 603–607. 10.1038/nature11003 22460905 PMC3320027

[B5] Chandrashekar D. S. Bashel B. Balasubramanya S. A. H. Creighton C. J. Ponce-Rodriguez I. Chakravarthi B. V. S. K. (2017). UALCAN: A Portal for Facilitating Tumor Subgroup Gene Expression and Survival Analyses. Neoplasia 19 (8), 649–658. 10.1016/j.neo.2017.05.002 28732212 PMC5516091

[B6] Chen P. Yue X. Xiong H. Lu X. Ji Z. (2019). RBM3 Upregulates ARPC2 by Binding the 3'UTR and Contributes to Breast Cancer Progression. Int. J. Oncol. 54 (4), 1387–1397. 10.3892/ijo.2019.4698 30720048

[B7] Cheng Z. Wei W. Wu Z. Wang J. Ding X. Sheng Y. (2019). ARPC2 Promotes Breast Cancer Proliferation and Metastasis. Oncol. Rep. 41 (6), 3189–3200. 10.3892/or.2019.7113 31002363 PMC6488984

[B8] Choi J. Lee Y. J. Yoon Y. J. Kim C. H. Park S. J. Kim S. Y. (2019). Pimozide Suppresses Cancer Cell Migration and Tumor Metastasis through Binding to ARPC2, a Subunit of the Arp2/3 Complex. Cancer Sci. 110 (12), 3788–3801. 10.1111/cas.14205 31571309 PMC6890432

[B9] Firat-Karalar E. N. Welch M. D. (2011). New Mechanisms and Functions of Actin Nucleation. Curr. Opin. Cel Biol. 23 (1), 4–13. 10.1016/j.ceb.2010.10.007 PMC3073586 21093244

[B10] García-Ponce A. Citalán-Madrid A. F. Velázquez-Avila M. Vargas-Robles H. Schnoor M. (2015). The Role of Actin-Binding Proteins in the Control of Endothelial Barrier Integrity. Thromb. Haemost. 113 (1), 20–36. 10.1160/th14-04-0298 25183310

[B11] Iwaya K. Norio K. Mukai K. (2007). Coexpression of Arp2 and WAVE2 Predicts Poor Outcome in Invasive Breast Carcinoma. Mod. Pathol. 20 (3), 339–343. 10.1038/modpathol.3800741 17277766

[B12] Kang M. Lu S. Kuan Chong P. Guan Yeoh K. Pin Lim Y. (2016). Comparative Proteomic Profiling of Extracellular Proteins between Normal and Gastric Cancer Cells. Curr. Cancer. Drug. Target. 16 (5), 442–454. 10.2174/1568009616666151209113606 26648486

[B13] Kinoshita T. Nohata N. Watanabe-Takano H. Yoshino H. Hidaka H. Fujimura L. (2012). Actin-related Protein 2/3 Complex Subunit 5 (ARPC5) Contributes to Cell Migration and Invasion and Is Directly Regulated by Tumor-Suppressive microRNA-133a in Head and Neck Squamous Cell Carcinoma. Int. J. Oncol. 40 (6), 1770–1778. 10.3892/ijo.2012.1390 22378351

[B14] Kiuchi T. Nagai T. Ohashi K. Mizuno K. (2011). Measurements of Spatiotemporal Changes in G-Actin Concentration Reveal its Effect on Stimulus-Induced Actin Assembly and Lamellipodium Extension. J. Cel Biol 193 (2), 365–380. 10.1083/jcb.201101035 PMC3080261 21502360

[B15] Laurila E. Savinainen K. Kuuselo R. Karhu R. Kallioniemi A. (2009). Characterization of the 7q21-Q22 Amplicon Identifies ARPC1A, a Subunit of the Arp2/3 Complex, as a Regulator of Cell Migration and Invasion in Pancreatic Cancer. Genes Chromosom. Cancer 48 (4), 330–339. 10.1002/gcc.20643 19145645

[B16] Li T. Fu J. Zeng Z. Cohen D. Li J. Chen Q. (2020). TIMER2.0 for Analysis of Tumor-Infiltrating Immune Cells. Nucleic Acids Res. 48 (W1), W509–W514. 10.1093/nar/gkaa407 32442275 PMC7319575

[B17] Lin S. Zheng L. Lu Y. Xia Q. Zhou P. Liu Z. (2020). Comprehensive Analysis on the Expression Levels and Prognostic Values of LOX Family Genes in Kidney Renal clear Cell Carcinoma. Cancer Med. 9 (22), 8624–8638. 10.1002/cam4.3472 32970930 PMC7666732

[B18] Liu Z. Yang X. Chen C. Liu B. Ren B. Wang L. (2013). Expression of the Arp2/3 Complex in Human Gliomas and its Role in the Migration and Invasion of Glioma Cells. Oncol. Rep. 30 (5), 2127–2136. 10.3892/or.2013.2669 23969835

[B19] Molinie N. Gautreau A. (2018). The Arp2/3 Regulatory System and its Deregulation in Cancer. Physiol. Rev. 98 (1), 215–238. 10.1152/physrev.00006.2017 29212790

[B20] Moriya Y. Nohata N. Kinoshita T. Mutallip M. Okamoto T. Yoshida S. (2012). Tumor Suppressive microRNA-133a Regulates Novel Molecular Networks in Lung Squamous Cell Carcinoma. J. Hum. Genet. 57 (1), 38–45. 10.1038/jhg.2011.126 22089643

[B21] Pollard T. Beltzner C. C. (2002). Structure and Function of the Arp2/3 Complex. Curr. Opin. Struct. Biol. 12 (6), 768–774. 10.1016/s0959-440x(02)00396-2 12504682

[B22] Rauhala H. E. Teppo S. Niemelä S. Kallioniemi A. (2013). Silencing of the ARP2/3 Complex Disturbs Pancreatic Cancer Cell Migration. Anticancer Res. 33 (1), 45–52 . 23267127

[B23] Rhodes D. R. Kalyana-Sundaram S. Mahavisno V. Varambally R. Yu J. Briggs B. B. (2007). Oncomine 3.0: Genes, Pathways, and Networks in a Collection of 18,000 Cancer Gene Expression Profiles. Neoplasia 9 (2), 166–180. 10.1593/neo.07112 17356713 PMC1813932

[B24] Rotty J. D. Brighton H. E. Craig S. L. Asokan S. B. Cheng N. Ting J. P. (2017). Arp2/3 Complex Is Required for Macrophage Integrin Functions but Is Dispensable for FcR Phagocytosis and *In Vivo* Motility. Develop. Cel 42 (5), 498–513. 10.1016/j.devcel.2017.08.003 PMC5601320 28867487

[B25] Semba S. Iwaya K. Matsubayashi J. Serizawa H. Kataba H. Hirano T. (2006). Coexpression of Actin-Related Protein 2 and Wiskott-Aldrich Syndrome Family Verproline-Homologous Protein 2 in Adenocarcinoma of the Lung. Clin. Cancer Res. 12 (8), 2449–2454. 10.1158/1078-0432.ccr-05-2566 16638851

[B26] Su X. Wang S. Huo Y. Yang C. (2018). Short Interfering RNA-Mediated Silencing of Actin-Related Protein 2/3 Complex Subunit 4 Inhibits the Migration of SW620 Human Colorectal Cancer Cells. Oncol. Lett. 15 (3), 2847–2854. 10.3892/ol.2017.7642 29435011 PMC5778834

[B27] Tan C. K. Law N. M. Ng H. S. Machin D. (2003). Simple Clinical Prognostic Model for Hepatocellular Carcinoma in Developing Countries and its Validation. J Clin. Oncol. 21 (12), 2294–2298. 10.1200/jco.2003.03.151 12805329

[B28] Tang Z. Li C. Kang B. Gao G. Li C. Zhang Z. (2017). GEPIA: a Web Server for Cancer and normal Gene Expression Profiling and Interactive Analyses. Nucleic Acids Res. 45 (W1), W98–W102. 10.1093/nar/gkx247 28407145 PMC5570223

[B29] Thul P. J. Akesson L. Wiking M. Mahdessian D. Geladaki A. Ait Blal H. (2017). A Subcellular Map of the Human Proteome. Science 356 (6340). eaal3321. 10.1126/science.aal3321 28495876

[B30] Tomczak K. Czerwinska P. Wiznerowicz M. (2015). The Cancer Genome Atlas (TCGA): an Immeasurable Source of Knowledge. Contemp. Oncol. (Pozn) 19 (1A), A68–A77. 10.5114/wo.2014.47136 25691825 PMC4322527

[B31] Tur-Gracia S. Martinez-Quiles N. (2021). Emerging Functions of Cytoskeletal Proteins in Immune Diseases. J. Cel Sci 134 (3). jcs253534. 10.1242/jcs.253534 33558442

[B32] Villanueva A. (2019). Hepatocellular Carcinoma. N. Engl. J. Med. 380 (15), 1450–1462. 10.1056/nejmra1713263 30970190

[B33] Xiong T. Luo Z. (2018). The Expression of Actin-Related Protein 2/3 Complex Subunit 5 (ARPC5) Expression in Multiple Myeloma and its Prognostic Significance. Med. Sci. Monit. 24, 6340–6348. 10.12659/msm.908944 30201948 PMC6144731

[B34] Xu N. Qu G. Y. Wu Y. P. Lin Y. Z. Chen D. N. Li X. D. (2020). ARPC4 Promotes Bladder Cancer Cell Invasion and Is Associated with Lymph Node Metastasis. J. Cel Biochem 121 (1), 231–243. 10.1002/jcb.29136 31190401

[B35] Zhang J. Liu Y. Yu C. J. Dai F. Xiong J. Li H. J. (2017). Role of ARPC2 in Human Gastric Cancer. Mediators Inflamm. 2017, 5432818. 10.1155/2017/5432818 28694563 PMC5485321

[B36] Zhang Y. Shen H. Liu H. Feng H. Liu Y. Zhu X. (2017). Arp2/3 Complex Controls T Cell Homeostasis by Maintaining Surface TCR Levels via Regulating TCR(+) Endosome Trafficking. Sci. Rep. 7 (1), 8952. 10.1038/s41598-017-08357-4 28827576 PMC5566485

[B37] Zhu A. X. Finn R. S. Edeline J. Cattan S. Ogasawara S. Palmer D. (2018). Pembrolizumab in Patients with Advanced Hepatocellular Carcinoma Previously Treated with Sorafenib (KEYNOTE-224): a Non-randomised, Open-Label Phase 2 Trial. Lancet Oncol. 19 (7), 940–952. 10.1016/S1470-2045(18)30351-6 29875066

